# Mitochondrial Dysfunction in the Inflammatory Process of Neurodegenerative Diseases

**DOI:** 10.3390/biomedicines14030682

**Published:** 2026-03-16

**Authors:** Salvatore Nesci

**Affiliations:** Department of Veterinary Medical Sciences, University of Bologna, 40064 Ozzano Emilia, Italy; salvatore.nesci@unibo.it

**Keywords:** mitochondrial dysfunction, oxidative stress, inflammation, respiratory complexes

## Abstract

Neurodegenerative diseases share a mitochondrial–immune axis in which impaired oxidative phosphorylation reshapes neuronal metabolism and drives chronic inflammation. Complex I play a redox gatekeeper role at the coenzyme Q (CoQ) junction: catalytic defects, misassembly, or reverse electron transport over-reduce the CoQ pool, increase electron leak, and elevate ROS. How respiratory supercomplex plasticity (CI-CIII_2_, CIII_2_-CIV_n_, or CI-CIII_2_-CIV_n_) modulates carrier channelling, flux control, and ROS propensity through dynamic reorganization of the electron transport chain is highlighted. Excess ROS damages lipids and mitochondrial DNA, promoting the release of mitochondrial damage-associated molecular patterns s that activate NLRP3 inflammasome signalling, cGAS-STING-dependent interferon programs, and endosomal TLR9 pathways, establishing feed-forward loops between mitochondrial injury and neuroinflammation. Disease-focused sections integrate evidence from Parkinson’s, Alzheimer’s, amyotrophic lateral sclerosis, and Huntington’s models, and map these mechanisms onto therapeutic opportunities spanning electron transport chain support, supercomplex stabilization, and consider mtDNA-sensing inflammatory nodes.

## 1. Introduction

**Figure 1:**
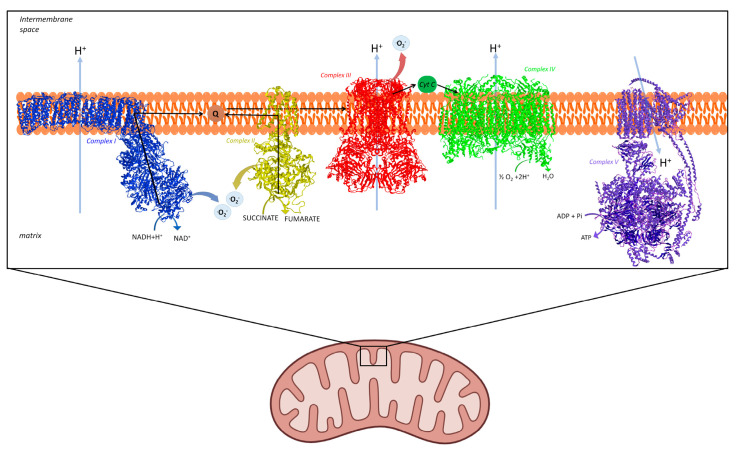
Oxidative phosphorylation in mitochondria. Electron transfer through complexes I–IV pumps protons across the inner mitochondrial membrane, creating the gradient that powers ATP synthesis by complex V. The site of ROS production are highlighted. Complex I, Complex II, Complex III, Complex IV, and Complex V are shown in their free form modified PDB IDs: 6YJ4, 1ZOY, 2YBB, 1V54, and 6TT7, respectively.

Mitochondria are central regulators of neuronal survival because they integrate ATP production, redox homeostasis, calcium buffering, and stress signaling. In neurodegenerative diseases, including Parkinson’s disease (PD), Alzheimer’s disease (AD), amyotrophic lateral sclerosis (ALS), and Huntington’s disease (HD), convergent evidence implicates impaired oxidative phosphorylation (OXPHOS) as a driver of synaptic failure and progressive neuronal loss [1]. Disruption of electron flow through the respiratory chain reduces ATP availability and increases the probability of electron leak to oxygen, generating reactive oxygen species (ROS). Although low ROS levels support adaptive redox signalling, sustained or compartmentally dysregulated ROS promotes oxidative damage to lipids, proteins, and nucleic acids and establishes feed-forward cycles that further impair mitochondrial bioenergetics [2] (Figure 1).

Among OXPHOS components, Complex I (NADH:ubiquinone oxidoreductase) is a major hub linking altered catalysis to oxidative stress. Complex I dysfunction can arise from changes in subunit composition, post-translational modification, oxidative damage, or misassembly, shifting the redox poise of NADH/NAD^+^ and the ubiquinone pool and prolonging the lifetime of reduced redox intermediates that can donate electrons to oxygen. In PD, biochemical studies of human brain mitochondria show that Complex I contains oxidatively damaged catalytic subunits and exhibits impaired function associated with misassembly, supporting a disease-relevant connection between Complex I catalytic integrity and oxidative stress [3]. In addition to increased ROS during forward electron transfer under pathological inhibition or damage, reverse electron transport (RET), favoured by a highly reduced Coenzyme Q (CoQ) pool, elevated membrane potential in the presence of succinate, can drive particularly high superoxide production at Complex I, highlighting how changes in electron flux directionality can convert metabolic states into oxidative stress [4].

A further layer of regulation is provided by the supramolecular organization of the respiratory chain into supercomplexes (SCs). Dynamic assembly models propose that respiratory complexes can exist in both free and super-assembled states, allowing flexible optimization of electron transfer under changing metabolic demands [5,6]. Altered supercomplex stability has been proposed to influence electron transfer efficiency, complex stability, and the propensity for ROS generation, although the field continues to debate the extent to which supercomplexes promote substrate channelling versus serving primarily structural and regulatory roles [7,8]. In neurodegenerative contexts, oxidative damage to inner membrane lipids (e.g., cardiolipin), altered proteostasis, and Complex I injury may promote supercomplex disassembly, increasing exposure of redox centers and favouring inefficient electron transfer states that elevate ROS.

Importantly, mitochondrial ROS are not only toxic byproducts but also signals that couple bioenergetic dysfunction to neuroinflammation. Oxidative stress can promote mitochondrial damage and the release of mitochondrial damage-associated molecular patterns (mtDAMPs), including oxidized mitochondrial DNA (mtDNA), which activate innate immune pathways such as the NLRP3 inflammasome and amplify inflammatory cytokine production [9,10]. This creates a bidirectional loop in which inflammation further impairs mitochondrial function and antioxidant capacity, reinforcing oxidative stress and accelerating neurodegenerative progression. Taken together, Complex I catalytic alterations, supercomplex remodelling, and ROS-driven inflammatory signalling represent an interconnected pathogenic axis and a set of tractable targets for therapies aimed at restoring mitochondrial efficiency, redox balance, and immune homeostasis.

## 2. Respiratory Supercomplex Plasticity Shapes Complex I Redox Behaviour and ROS Generation

Oxidative stress emerging from altered Complex I activity can be framed as a problem of flux control at the CoQ junction coupled to changes in SC organization. In the “plasticity” view, SC assembly/disassembly dynamically tunes electron transfer efficiency and can help minimize electron leak, whereas SC destabilization can erode pathway robustness and increase ROS propensity, especially when electron supply and demand become mismatched [11,12]. Mechanistically, this can be understood through the idea that SCs can impose partial channeling/segmentation of electron carriers, including functionally dedicated CoQ pools, thereby shaping how NADH-derived electrons from Complex I and FADH_2_-derived electrons converge on ubiquinone and flow onward through Complex III/IV [6,11,13]. Consistent with this, genetic perturbation of SC interactions shows that SC assembly can define dedicated CoQ and cytochrome *c* pools and thereby organize electron flux to optimize substrate use [14]. When SCs disassemble or remodel, this “wiring” is weakened, and control of the CoQ redox state becomes more vulnerable to overload: excessive reduction in the CoQ pool (high CoQH_2_/CoQ) increases the thermodynamic drive for RET at Complex I, a condition associated with high superoxide output and redox signalling that can become maladaptive in chronic stress [4]. Importantly, the CoQH_2_/CoQ ratio itself functions as a sensor of respiratory chain efficiency, and when FAD-linked influx saturates CoQ oxidation capacity, RET-linked ROS can trigger Complex I destabilization/degradation and supercomplex reconfiguration, effectively rebalancing the ETC toward the prevailing substrate profile, but at the cost of oxidative stress when sustained [15]. Thus, Complex I sits at a critical control point where it both sets the redox poise of the CoQ pool during forward electron transfer and responds to CoQ over-reduction via RET, while SC integrity modulates whether electrons are efficiently “channelled” through the chain or diverted into ROS-generating states [11,14].

In mammalian mitochondria, the respiratory chain can adopt several recurrent supercomplex (SC) architectures, most commonly built around three “modules”: CI-CIII_2_-CIV. Early Blue-Native work and subsequent structural/functional studies support that CI is frequently found associated with CIII_2_, with variable incorporation of CIV, rather than existing only as fully free complexes. Three major assemblies are often discussed: CI-CIII_2_, CI-CIII_2_-CIV (the canonical “respirasome”), and CIII_2_-CIV [16,17].

This assembly links NADH oxidation by CI directly to CoQ reduction and oxidation at CIII_2_, placing CI and CIII_2_ in proximity. Functionally, CI-CIII_2_ is frequently viewed as (i) a stabilizing environment for CI and/or (ii) a configuration that can bias electron flux by shaping how electrons entering from NADH access the CoQ region and then proceed to cytochrome *c* via CIII. It is also a common “core” unit that can be extended by CIV addition to form respirasomes [14,16].

The respirasome is the best-known SC because it contains the full set of catalytic steps needed to transfer electrons from NADH to O_2_ (CI → CoQ → CIII → cyt *c* → CIV), making it the simplest structural entity capable of “self-contained” NADH-supported respiration. However, the CIV stoichiometry in respirasome CI-CIII_2_-CIV_n_ can vary (as IV_1–4_ depending on species/tissue/detergent conditions) [17]. Importantly, SC formation is not necessarily a single end-state: supercomplex assembly is dynamic and can reorganize electron flux to match substrate availability. Beyond post-assembly association models, respirasome biogenesis can involve a CI assembly intermediate acting as a scaffold for incorporation of CIII/CIV subunits, supporting “cooperative assembly” concepts [18].

A distinct family of SCs is formed by CIII_2_ associated with CIV. This unit is often referred to as a Q-respirasome because it is positioned to oxidize CoQH_2_ generated by FAD-linked inputs (e.g., CII, ETF:Q oxidoreductase, glycerol-3-phosphate dehydrogenase), passing electrons from CoQ → CIII → cyt *c* → CIV to reduce oxygen. In mammals, formation of the CIII_2_-CIV interaction is strongly influenced by the supercomplex assembly factor *SCAF1*/*COX7A2L*, providing genetic/structural evidence for regulated CIII_2_-CIV super-assembly [12,19,20,21].

## 3. Bidirectional Crosstalk Between Mitochondrial Dysfunction and Neuroinflammation in Neurodegeneration

When OXPHOS is compromised, the same respiratory machinery that normally couples electron transfer to proton pumping can become a dominant generator of ROS, shifting mitochondria from efficient bioenergetic hubs into sources of redox injury and inflammatory signalling. Under physiological conditions, electron flow through the electron transport chain (ETC) builds the proton motive force (pmf) that drives ATP synthase, while mitochondrial ROS are kept low enough to support adaptive signalling [2]. In neurodegenerative settings, however, defects in ETC complexes, substrate overload, impaired ADP/ATP exchange, or ATP synthesis can increase pmf while restricting ATP production, a state that favours electron leak from redox centers and elevates superoxide and downstream oxidants. Persistent ROS overproduction damages mitochondrial lipids and proteins and is particularly mutagenic for mtDNA, which is located near ETC-derived oxidants and relies on relatively limited repair capacity compared with nuclear DNA [22]. As mtDNA becomes oxidized, fragmented, or improperly packaged, mitochondrial membranes can become permeabilized through stress-associated pores, apoptosis-related outer membrane permeabilization, or defective mitophagy, allowing mtDNA and other mitochondrial constituents to escape into the cytosol or extracellular space. These released molecules function as mitochondrial damage-associated molecular patterns (mtDAMPs), including mtDNA, cardiolipin, and oxidized proteins, that are interpreted by the cell as “danger” signals capable of initiating innate immune responses [23]. A central node in this process is the NLRP3 inflammasome: damaged, ROS-generating mitochondria accumulate when mitophagy is impaired, and mitochondrial ROS acts as a permissive signal for NLRP3 activation, linking mitochondrial quality control failure to caspase-1 activation and maturation of IL-1β/IL-18 [10]. Beyond ROS as a trigger, mtDNA itself can directly contribute to inflammasome activation; oxidized mtDNA released during mitochondrial stress can bind and activate NLRP3, providing a mechanistic bridge between oxidative damage and inflammatory execution pathways [24,25]. Beyond NLRP3, other inflammasomes may also intersect with mitochondrial injury in neuroinflammatory settings. Among them, Absent in melanoma 2 (AIM2) currently has the strongest mechanistic link to mitochondrial damage, as misplaced or released mitochondrial DNA can promote AIM2 activation; this mtDNA-AIM2 axis has been demonstrated experimentally and AIM2 has also been implicated in AD and PD models [26,27], with a recent dedicated review summarizing its role in neurodegenerative disorders [28]. Evidence for NLRC4 is more limited but suggests that mitochondrial damage and mtDNA release can facilitate NLRC4 activation, while sterile CNS danger signals can activate NLRC4 in microglia and astrocytes [29]. By contrast, NLRP6 seems to have a more context-dependent and still less well-defined role in the nervous system; current studies support links to neuroinflammatory regulation and mitochondrial homeostasis rather than a firmly established mtDNA-sensing pathway in classical neurodegenerative disease [30]. Overall, the non-NLRP3 literature is strongest for AIM2, whereas NLRC4 and NLRP6 should be presented as emerging pathways that warrant further validation in AD, PD, ALS, and HD.

In parallel, cytosolic mtDNA is sensed by DNA surveillance pathways such as cGAS-STING, which drives IRF3-dependent transcriptional programs including type I interferon and inflammatory mediators; importantly, mtDNA “stress” caused by altered mtDNA packaging can promote mtDNA escape into the cytosol and engage cGAS-STING signaling, demonstrating that mtDNA instability is itself an innate immune trigger [31,32]. These inflammatory outputs are not confined to peripheral immunity; within the central nervous system, microglia and astrocytes respond vigorously to DAMPs and redox cues, amplifying cytokine production and reshaping synaptic homeostasis. Thus, mitochondrial dysfunction can convert a metabolic defect into a neuroimmune phenotype: ROS injures mtDNA and membranes, mtDAMPs leak, and cytosolic sensors translate this “misplaced self” into inflammasome activation and interferon/cytokine cascades [23]. This sequence is highly relevant to neurodegenerative diseases, where both mitochondrial abnormalities and chronic neuroinflammation are recurring, mutually reinforcing hallmarks. In PD, for example, defects in mitochondrial quality control pathways converge with inflammatory signalling; impaired mitophagy increases the persistence of damaged, ROS-producing mitochondria, raising the probability of mtDNA release and innate immune activation. The broader concept that mtDNA release and cytosolic DNA sensing can couple mitochondrial stress to inflammation is increasingly used to interpret why neurodegeneration often tracks with prolonged microglial activation and cytokine elevation [23]. Similar logic extends to AD and other proteinopathies, where oxidative stress, mitochondrial injury, and inflammatory mediators co-evolve: inflammatory signalling can impair mitochondrial respiration and antioxidant buffering, while mitochondrial ROS and mtDAMPs sustain glial activation, creating a feed-forward loop that accelerates synaptic dysfunction and neuronal vulnerability. In this framework, mitochondria are not passive casualties of disease but active organizers of pathological crosstalk between metabolism and immunity: they determine whether ETC stress is resolved through quality control and adaptive redox signalling or escalates into mtDNA damage, DAMP release, innate immune activation, and chronic neuroinflammation. Ultimately, the progression of neurodegenerative diseases can be viewed as a failure to contain mitochondrial stress, where disrupted SC organization and ROS excess not only deprive neurons of energy but also initiate and perpetuate inflammatory programs that magnify tissue damage, underscoring mitochondria as critical drivers, biomarkers, and therapeutic targets in neurodegeneration [23].

Extracellular vesicles (EVs) carrying mitochondrial cargo, including mtDNA, respiratory-chain proteins, cardiolipin, and even mitochondrial fragments, may represent a promising minimally invasive biomarker platform in neurodegenerative diseases. Recent reviews focused on the nervous system explicitly highlight the potential diagnostic and prognostic value of mitochondrial EVs, while also noting that EV-associated mitochondrial DAMPs may actively contribute to early neuroinflammation rather than merely reflect tissue damage [33].

Complex I dysfunction can be mechanistically linked to supercomplex disassembly, ROS escalation, and mtDNA-driven inflammation through a self-reinforcing sequence of structural and redox events. When Complex I catalytic activity is impaired or forced into inefficient electron-transfer states, electrons accumulate at upstream redox centers and the CoQ pool becomes abnormally reduced, increasing the probability of electron leak and superoxide formation, particularly under conditions that favour RET [34]. Elevated ROS then oxidizes cardiolipin and other inner membrane components that help stabilize respiratory assemblies, weakening the physical integrity of Complex I-containing SCs (e.g., CI-CIII_2_ and CI-CIII_2_-CIV_n_) and shifting the ETC toward less coordinated electron transfer with poorer flux control [8,14]. As supercomplex architecture destabilizes, Complex I becomes more prone to misassembly from SCs and electron transfer becomes “leakier,” further elevating ROS and amplifying oxidative damage to mitochondrial proteins, lipids, and mtDNA [35]. ROS-injured mitochondria are then more likely to undergo permeability transitions or incomplete quality control, enabling mtDNA to escape into the cytosol or extracellular milieu as a mitochondrial DAMP. Once mislocalized, mtDNA is recognized by innate immune sensors that convert mitochondrial damage into inflammation, including NLRP3 and cGAS-STING, thereby coupling Complex I/SCs dysfunction directly to inflammatory signalling and cell injury [36].

Emerging studies have also highlighted the mitochondrial-lysosomal axis as an important pathogenic mechanism across neurodegenerative disorders. Their dysregulation is increasingly implicated in neuronal homeostasis failure and disease progression [37]. This appears especially relevant in PD with disruption of mitochondrialysosome contacts in human dopaminergic neurons [38], and in microglial systems, where lysosomal acidification defects are increasingly linked to impaired mitophagy/autophagy, persistent inflammatory activation, and neurodegenerative progression [39].

## 4. From Mitochondrial Stress to Neurodegeneration: The Inflammatory Cascade

**Figure 2:**
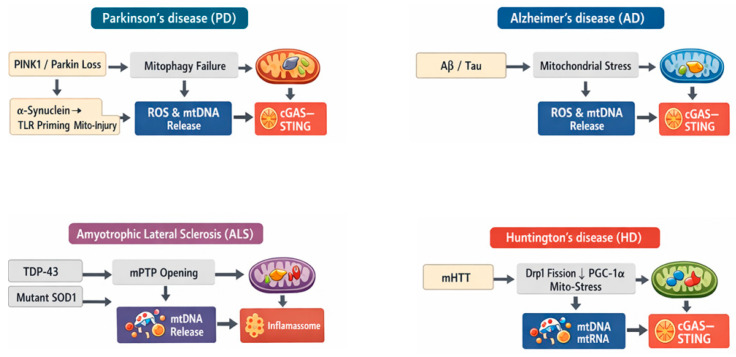
Disease-specific triggers converging on mito-inflammation. Four comparable panels depict how disease-linked mitochondrial defects promote mtDNA/ROS-dependent activation of innate immune pathways. PD: PINK1/Parkin mitophagy failure and α-synuclein-driven TLR priming/mitochondrial injury activate NLRP3 and cGAS-STING. AD: Aβ/tau-induced mitochondrial stress triggers cGAS-STING and NLRP3, amplified by microglia. ALS: TDP-43–induced mPTP opening releases mtDNA to engage cGAS-STING, while mutant SOD1 promotes inflammasome activation. HD: mHTT-driven Drp1 fission and PGC-1α suppression increase mitochondrial stress and nucleic-acid sensing/inflammasome signaling. Common outputs include type I interferons, IL-1β/IL-18, and glial activation that exacerbate neurodegeneration.

Mitochondrial dysfunction can exacerbate uncontrolled innate immune responses and cell death by converting “misplaced self” mitochondrial nucleic acids into potent inflammatory triggers. When oxidative stress and membrane damage promote mtDNA leakage into the cytosol, cytosolic DNA sensing via the cGAS-STING axis can drive sustained type I interferon and NF-κB-linked cytokine programs, reinforcing neuroinflammation and sensitizing neurons to degeneration [40]. In parallel, damaged mitochondria amplify inflammasome activation: mitochondrial ROS and the release of oxidized mtDNA can activate NLRP3, promoting caspase-1 activation, IL-1β/IL-18 maturation, and inflammatory pyroptosis-like outcomes that intensify bystander injury in the neural microenvironment [41]. A complementary pathway operates in endolysosomes, where mtDNA rich in CpG motifs can engage TLR9, a mechanism demonstrated in sterile inflammation when mtDNA escapes and triggers TLR9-dependent cytokine signalling [42]. In the central nervous system, TLR9 stimulation in microglia has been linked to the release of neurotoxic mediators (e.g., TNF-α, NO), providing a plausible route by which mtDNA-driven TLR9 signalling can worsen neuronal injury [43]. Together, activation of cGAS-STING, NLRP3, and TLR9 establishes a feed-forward process in which mitochondrial damage produces inflammation that further impairs mitochondrial homeostasis, and the combined stress accelerates tissue injury and neurodegenerative disease. Distinct neurodegenerative diseases can converge on common mitochondrial danger-signaling pathways that promote neuroinflammation and cellular injury (Figure 2).

### 4.1. Mitochondrial Dysfunction and Pattern Recognition Receptor Activation in Parkinson’s Disease

A concrete PD example that connects mitochondrial dysfunction-innate immune overactivation-neurodegeneration is the PINK1/Parkin-mtDNA-cGAS-STING (“mitoflammation”) axis. PINK1-Parkin-mediated mitochondrial quality control (mitophagy) is thought to limit mitochondrial stress and restrain the aberrant cytosolic escape of mtDNA, which otherwise can act as a DAMP and activate the DNA-sensing cGAS-STING pathway, promoting type I interferon/NF-κB-linked inflammatory signaling relevant to Parkinson’s disease [44]. Accordingly, mitochondrial dysfunction and mtDNA release are frequently discussed as upstream triggers of cGAS-STING-driven neuroinflammation in PD, even though the strength and context-dependence of this axis can vary across models and cell types [45]. In parallel, a complementary PD-relevant mechanism links the PD proteinopathy to inflammasome activation: extracellular α-synuclein fibrils can be taken up by microglia and drive NLRP3 inflammasome assembly, resulting in caspase-1 activation and IL-1β release [46]. Mechanistically, misfolded or aggregated α-synuclein delivers a priming signal through TLR2, leading to NF-κB-dependent induction of inflammasome components. Subsequent mitochondrial damage associated with α-synuclein internalization provides the activation signal that drives NLRP3-dependent cytokine maturation. In this context, antibody-α-synuclein immune complexes can simultaneously engage TLR2 and induce mitochondrial injury, thereby promoting full NLRP3 activation [47]. A third PD-specific illustration of innate immune amplification involves endolysosomal DNA sensing: microglial TLR9 activation (regulated by microglial glucocorticoid receptor (GR) signalling) was shown to be detrimental for dopaminergic neuron survival in the substantia nigra in settings triggered by TLR9 agonists, and human PD tissue showed reduced microglial GR consistent with sensitization to this pathway [48]. Together, these mechanisms exemplify how mitochondrial injury and PD-linked stressors can converge on cGAS-STING (interferon/cytokine transcription), NLRP3 (IL-1β/IL-18 maturation and inflammatory cell death programs), and TLR9 (DNA-driven microglial activation) to amplify neuroinflammation and accelerate dopaminergic neurodegeneration. Microglia are the best-supported effector cells in the pathways of PD neuroinflammation.

### 4.2. Mitochondrial Dysfunction as a Catalyst of Innate Immune Amplification in Alzheimer’s Disease

In AD models, Aβ-associated cellular stress can drive mitochondrial dysfunction and DNA damage, increasing the likelihood that mtDNA fragments accumulate outside mitochondria; in the 5×FAD model and in human AD brain, microglia show evidence of cytosolic double-stranded DNA binding to cGAS and activation of the cGAS-STING pathway, and genetic loss of cGAS (or pharmacologic STING inhibition) reduces neuroinflammation, amyloid pathology, and cognitive impairment [49]. Microglia exhibit the strongest and most direct innate-immune amplification receptors of mitochondrial stress in AD. In parallel, Aβ deposition engages NLRP3 inflammasome signalling in vivo: deletion of Nlrp3 or Casp1 in APP/PS1 mice protects against memory deficits and is associated with reduced IL-1β/caspase-1 activation and improved Aβ clearance [50]. Mechanistically, mitochondrial damage provides a biochemical route to inflammasome activation because oxidized mtDNA released during stress can bind and activate NLRP3, directly linking oxidative injury to inflammatory cytokine maturation [24]. Finally, endolysosomal DNA sensing can modulate AD pathology: stimulating TLR9 with CpG oligodeoxynucleotides in Tg2576 mice markedly reduces amyloid burden and improves behaviour, highlighting that DNA-sensing pathways in brain-resident immune cells can reshape disease trajectories [51]. These findings suggest that DNA-sensing pathways can exert context-dependent effects in AD, with transient activation by synthetic CpG ligands potentially driving protective microglial responses and amyloid clearance, whereas persistent endogenous mtDNA signaling is more often linked to detrimental neuroinflammation [51]. Together, these findings support a disease-relevant sequence in which Aβ-associated mitochondrial stress promotes mtDNA mislocalization and redox injury, which is then “translated” into chronic neuroinflammation through cGAS-STING and NLRP3, ultimately amplifying synaptic dysfunction and neurodegeneration.

### 4.3. Translating Mitochondrial Stress into Chronic Neuroinflammation in Amyotrophic Lateral Sclerosis (ALS)

In ALS, a particularly clear mitochondria-to-immunity mechanism has been defined for TDP-43 proteinopathy: cytoplasmic TDP-43 can trigger mitochondrial permeability transition pore (mPTP)-dependent mtDNA release into the cytosol, which is then sensed by cGAS, activating STING to drive type-I interferon/NF-κB inflammatory programs. Notably, this pathway was linked to TDP-43–associated pathology and accompanied by elevated cGAMP in spinal cord samples from ALS patients [52]. In parallel, familial ALS models driven by mutant superoxide dismutase 1 (SOD1) illustrate how mitochondrial/oxidative stress feeds inflammasome signalling: mutant SOD1 activates an ASC-dependent inflammasome in microglia, leading to caspase-1 activation and IL-1β production, which can accelerate disease and represents a therapeutic node [53]. Downstream of this axis, inflammasome machinery is detectable in disease-relevant CNS cells: NLRP3 inflammasome components and IL-1β are reported in the spinal cord of SOD1 (G93A) mice and in human ALS tissue [54], and oxidative/nitrosative stress products such as peroxynitrite can act as triggers that amplify caspase-1/IL-1β signaling in SOD1 (G93A) microglial systems [55]. A further amplification route is plausible via TLR9, because CpG-rich mtDNA DAMPs that enter endolysosomal compartments can engage TLR9-driven inflammatory signalling; consistent with relevance to motor-neuron vulnerability, TLR9 antagonism has been shown to protect spinal cord neurons against excitotoxic death in vitro [56]. Together, these ALS-linked examples illustrate how mitochondrial injury and redox stress can be “translated” into chronic neuroinflammation through cGAS-STING and inflammasome pathways, with endosomal DNA sensing as a potential additional amplifier of tissue damage. In ALS, neurons initiate, microglia amplify, and astrocytes propagate mtDNA-derived innate immune signaling.

### 4.4. Mitochondrial Fragmentation–Driven Innate Immune Activation in Huntington’s Disease

In HD, mutant huntingtin (mHTT) couples mitochondrial damage to maladaptive innate immune activation through several converging molecular routes in vulnerable striatal neurons and glia. A key mitochondrial initiating event is abnormal mitochondrial fission: mHTT binds the fission GTPase Drp1, increases Drp1 enzymatic activity, and drives excessive mitochondrial fragmentation with impaired axonal trafficking, changes that are accompanied by mitochondrial respiration decline and enhanced oxidative stress vulnerability [57]. In parallel, mHTT suppresses mitochondrial homeostatic programs at the transcriptional level by repressing PGC-1α, reducing mitochondrial biogenesis and respiratory gene expression, and thereby weakening the cell’s capacity to maintain redox balance during energetic stress [58]. When these defects persist, damaged mitochondria become prone to releasing normally sequestered mitochondrial nucleic acids that act as innate immunogens. In fact, cell-type-resolved transcriptomics in HD revealed that striatal spiny projection neurons exhibit mitochondrial RNA (mtRNA) release and induction of neuronal innate immune signalling, with evidence that released mtRNAs can bind the innate immune sensor PKR, linking mitochondrial disruption to inflammatory stress pathways in precisely the cell population most susceptible in HD [59]. Beyond mitochondrial nucleic acids, mHTT can promote genome instability that further fuels cytosolic nucleic-acid sensing: mHTT-driven DNA damage and accumulation of cytosolic DNA activate the cGAS-STING pathway, engaging TBK1/STAT1 signalling and promoting apoptosis, providing a mechanistic bridge from mHTT-induced cellular stress to innate immune signalling and cell death [60]. Downstream, these innate immune cues amplify inflammatory effector pathways such as the NLRP3 inflammasome; consistent with a pathogenic role, chronic pharmacologic inhibition of NLRP3 with MCC950 in R6/2 HD mice suppresses inflammasome activation, lowers IL-1β and ROS, reduces gliosis, improves neuronal survival, and ameliorates motor decline [61]. Together, these findings indicate that astrocytes act as reactive responders, contributing to inflammation and neuronal stress but not the primary source of nucleic-acid-driven innate immune activation. They also outline a coherent HD mechanism in which mHTT-driven mitochondrial fragmentation and impaired bioenergetic/redox programming promote nucleic-acid-based danger signalling (mtRNA and cytosolic DNA), engage cGAS-STING and inflammasome cascades, and thereby lock mitochondrial dysfunction and neuroinflammation into a feed-forward loop that accelerates neurodegeneration.

## 5. Therapeutic Strategies to Interrupt Mitochondria-Driven Neuroinflammation

Potential therapeutic strategies for mitochondrial danger signaling in neurodegenerative disease can be grouped into three levels: (i) mitochondrial support to reduce ROS production, mtDNA leakage, and bioenergetic failure; (ii) blockade of upstream innate immune sensors such as cGAS-STING, NLRP3, and, context-dependently, TLR9; and (iii) suppression of downstream inflammatory effectors. In PD, nicotinamide riboside (NR) increased cerebral NAD-related metabolites in randomized early-phase studies [62,63], while direct inflammasome targeting has entered the clinic with the oral brain-penetrant NLRP3 inhibitor VENT-02 in mild-to-moderate PD. Preclinically, pharmacologic NLRP3 inhibition with OLT1177/dapansutrile reduced α-synuclein burden and protected dopaminergic neurons in PD models [64]. In AD, suppression of the cGAS-STING pathway and NAD^+^ replenishment reduced amyloid-linked neuroinflammation and cognitive decline in mouse models [49], supporting this pathway as a promising but still mainly preclinical target. In ALS, the mitochondria-stabilizing bile acid TUDCA showed encouraging earlier clinical data, and a phase III TUDCA-ALS study has been registered [65], although definitive efficacy remains unsettled. In HD, the NLRP3 inhibitor MCC950 improved survival, motor performance, and neuroinflammation in R6/2 mice [61]. Overall, the most advanced translational avenues currently appear to be mitoprotective metabolic support and NLRP3 inhibition, whereas direct cGAS-STING antagonists in AD, PD, ALS, and HD remain largely preclinical.

## 6. Conclusions

Neurodegenerative diseases converge on a shared pathogenic axis in which impaired OXPHOS, particularly Complex I dysfunction and SC remodelling, shifts mitochondria from efficient ATP production to electron leak and chronic ROS generation. ROS-driven damage to mitochondrial membranes and mtDNA promotes the release of mitochondrial DAMPs that engage innate immune sensors such as NLRP3, cGAS-STING, and TLR9, sustaining neuroinflammation and reinforcing mitochondrial failure in a feed-forward loop. This framework links disease-specific mechanisms across PD, AD, ALS, and HD to a common bioenergetics-redox-immunity cascade and highlights therapeutic opportunities at multiple nodes restoring ETC flux control and selectively dampening mtDNA-triggered inflammatory signalling. Longitudinal measurement of circulating cell-free mtDNA may offer a mechanistically grounded biomarker candidate to stratify patients and monitor treatment response, supporting mitochondria as both drivers and actionable targets in neurodegenerative progression.

## Data Availability

Not applicable.
